# From Signals to Events: A Maturity-Based Perspective on IoT-Enabled Process Mining

**DOI:** 10.3390/s26196293

**Published:** 2026-10-04

**Authors:** Tilen Tratnjek, Mateja Bule, Gregor Polančič

**Affiliations:** Faculty of Electrical Engineering and Computer Science, University of Maribor, Koroška Cesta 46, 2000 Maribor, Slovenia; mateja.bule@um.si (M.B.); gregor.polancic@um.si (G.P.)

**Keywords:** process maturity, process mining, Internet of things, process intelligence, event-creating, event-enriching

## Abstract

To bridge the gap between the digital and physical worlds, we need systems that can effectively transform reality into computable signals. This perspective paper examines key challenges in such transformation related to the gap between fine-grained sensor signals and activity-level events used in process intelligence tools. We examine these challenges along two perspectives. The first perspective considers two ways on how sensor data is entered into the event log—either sensor readings are abstracted to create new event information, or event information is already present, and sensor data enriches it with new context. The second perspective concerns the maturity of the operating process itself, considering how process maturity may influence the feasibility, reliability and potential usefulness of IoT integration. Integration is therefore not only a technical question of how sensor data becomes process events, but also a prior question of whether and when it is justified. We propose a conceptual perspective on this relationship and identify candidate assessment areas that could form a basis for a future IoT-enabled process mining maturity model; these areas are not empirically validated in this study. We close by considering auditable event logs as a basis for accountability as autonomous systems become more widespread.

## 1. Introduction

Internet of Things (IoT) is a network of physical objects equipped with sensors, software, and connectivity. These objects collect and exchange data over the internet, enabling us to bridge the gap between the physical and digital worlds by continuously translating real-world observations into digital signals. A common purpose of these objects is to transform everyday items into sources of real-time information that can be monitored, analysed, and acted upon. This allows organisations to track location, measure performance, detect anomalies, and predict failures as they occur. In doing so, the IoT can provide continuous streams of data that offer observations of how things function, rather than relying solely on how we assume they should [1,2].

As IoT spreads across industry, an increasing amount of physical-world data is generated each passing day. However, the true value of these devices does not lie in the data itself but in connecting data across systems and integrating different data streams to answer questions such as “what happened?”, “when did it happen?”, “why did it happen?”, and “how does it fit into the bigger picture?”. This trend will only intensify as autonomy spreads: general-purpose humanoids and specialised robots are set to become major producers of process-relevant data, and decisions increasingly shift from rule-based systems to sensor-driven autonomous ones [3,4]. An auditable record of process execution will become increasingly important to understanding what was done, when it occurred, and who or what was responsible.

**Figure 1:**
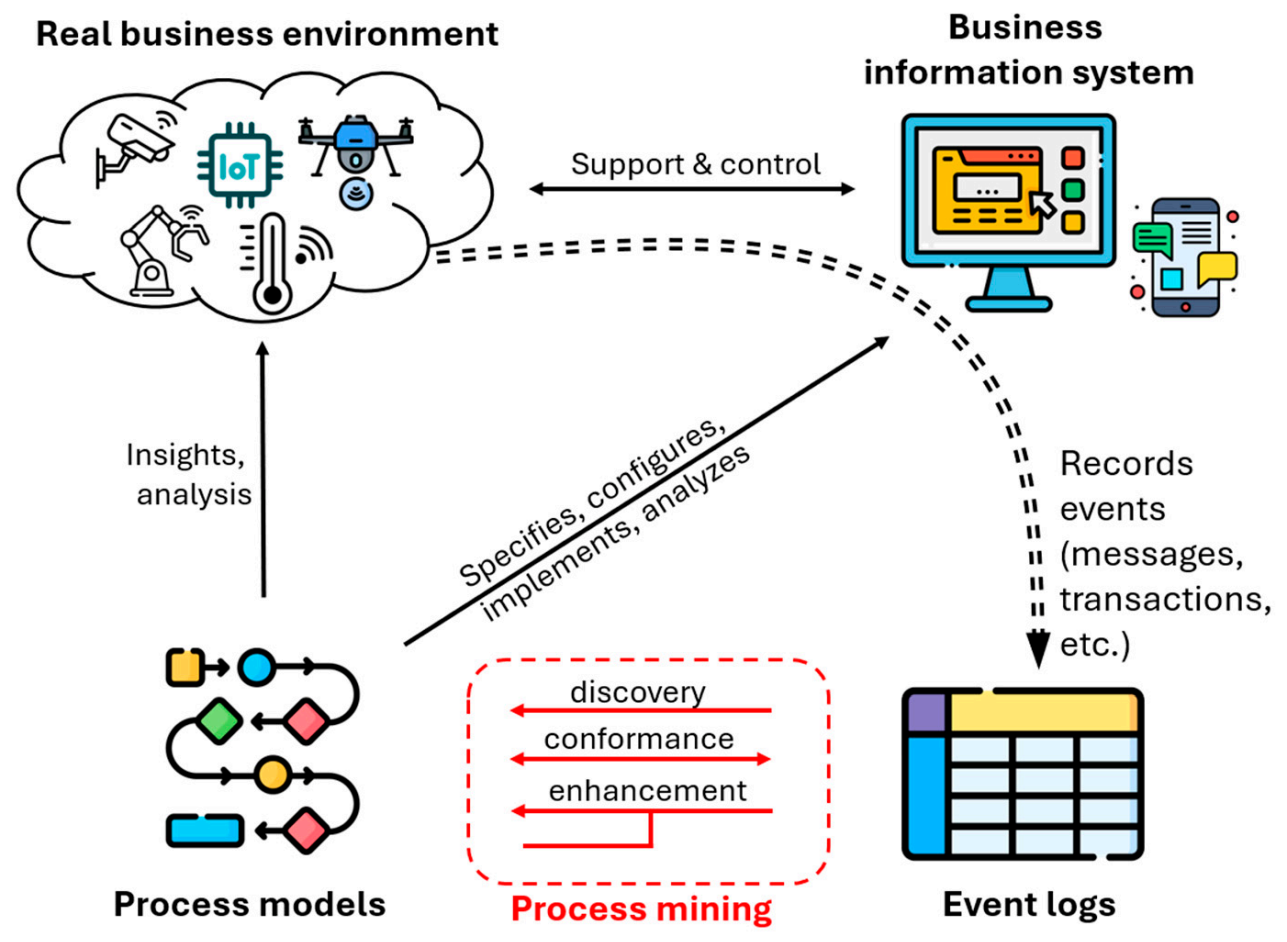
Process mining lifecycle and relationships between the components [5].

Process mining can be used to reconstruct, analyse, and improve operational processes by extracting insights from the digital traces recorded in event logs [5]. This provides three key capabilities, illustrated in Figure 1: discovering process models directly from logs; checking the conformity of modelled and observed behaviour; and enhancing existing models with additional perspectives, such as performance or resource data. These capabilities can help organisations identify where delays occur, which variants deviate from a reference process, and where potential compliance deviations occur, helping to replace subjective assumptions with empirically grounded evidence [5].

Taken together, the IoT and process mining can be mutually reinforcing; IoT brings real-world context to abstract process models [6], while process mining can give sensor data a clearer structure. As the reinforcement runs in both directions, and each direction poses a different challenge, we will consider them separately as follows:

- Contribution of Process Mining to the Internet of Things. IoT implementations are often designed to solve specific, isolated problems, generating vast streams of sensor data that lack business context and meaning [7]. Process mining can provide structure by relating sensor-derived observations to process activities. This allows organisations to understand how physical observations relate to process performance, transforming otherwise isolated sensor streams into process-relevant evidence [8].
- Contribution of Internet of Things to Process Mining. Process mining relies on event logs from information systems, which capture high-level business events but lack visibility into the physical execution of work. IoT can help address this gap by providing additional data from the physical world. This contextual enrichment can provide information not only about whether an event occurred, but also about the physical conditions under which it was executed, enabling forms of root-cause analysis that were previously difficult [7]. IoT-enriched events also enable more accurate predictions by feeding real-time physical conditions into predictive process models and support continuous compliance monitoring by flagging deviations as they happen [9,10].

The IoT-BPM manifesto by Janiesch et al. [11] highlights the potential benefits of combining the two paradigms while acknowledging the ongoing difficulty of transforming raw sensor signals into coherent and actionable process events. Although sensor-to-event abstraction is a recognised technical challenge, focusing on technical integration alone overlooks a more fundamental question: when does adding IoT data to process mining actually create sufficient value to justify the associated instrumentation, integration, governance, and analytical complexity? Addressing this question requires considering not only how sensor data can be transformed into events, but also what role IoT data play in process mining analysis, what additional insight they provide, and under which conditions their integration is worthwhile [12].

This paper therefore shifts the focus at the IoT–process mining intersection from whether sensor data can be technically integrated to when and how such integration is meaningful. In particular, we consider the role of IoT data, the process conditions under which it can be used reliably, and the capabilities required to support its use. As discussed in Section 2.3, existing research addresses important aspects of the relationship between IoT and process mining, particularly the transformation of sensor observations into process events and the development of process-mining capabilities. However, these perspectives have largely been considered independently, leaving unclear how the role of IoT data in the event log interacts with the maturity of the observed process. We bring these two perspectives together by distinguishing between event-creating and event-enriching IoT data and by considering process maturity as a contextual perspective that influences the feasibility, reliability, and potential usefulness of these two forms of integration. On this basis, the paper develops a perspective on the conditions, benefits, risks, prerequisites, and validation requirements associated with IoT-enabled process mining. The contribution is a perspective that connects the role of sensor data in constructing process knowledge with the maturity of the process and data environment in which that knowledge is generated.

The remainder of this paper is organised as follows: Section 2 reviews the relevant background, covering what process mining reads and produces and how sensor signals are transformed into process events. Section 3 develops a perspective on the role of IoT data in the event log, distinguishing between event-creating data and event-enriching data. Section 4 develops a perspective on process maturity, considering it as a contextual dimension that influences the feasibility, reliability, and potential usefulness of IoT-enabled process mining. Section 5 builds on these two perspectives by identifying candidate assessment areas and considerations that could provide a conceptual basis for the future development and empirical validation of such a maturity model. Section 6 discusses the relationship between the two perspectives, identifies a structured space of open topics and questions arising from their interaction, and concludes with an outlook on accountability as autonomous, sensor-driven systems become more prominent in organisational processes.

## 2. Background

Process mining relies on event logs to reconstruct and analyse how processes are actually executed in practice. While traditional event logs primarily capture digital interactions recorded by information systems, the increasing availability of IoT technologies provides an opportunity to extend this view with observations from the physical world. Sensors can capture conditions, movements, states, and other physical signals that may either generate previously unrecorded process events or provide additional context to events already present in the log [6,9].

### 2.1. Process Mining—What It Reads and What It Produces?

The event log is an essential component of process mining: it is a structured record in which each row carries, at a minimum, a case identifier, an activity label, and a timestamp. A case is one instance of the process, and the sequence of activities observed for a case is its variant. Real-world logs rarely contain one variant; they contain hundreds, and much of the analytical craft of process mining lies in deciding which variants represent the process and which represent noise, exceptions, or data-quality artefacts [5]. Figure 1 illustrates the continuous, cyclical nature of the process mining lifecycle and its core components. The cycle begins in the real business environment, where actual operational activities take place. These activities are recorded and digitised by the process-aware information system, generating event logs that capture specific actions, messages, and transactions. Through the core process mining techniques of discovery, conformance checking, and enhancement, these raw logs are transformed into visual process models. Finally, these models provide insights that feed back into the real business environment, completing the loop. The findings can also be used to configure and improve the process-aware information system.

**Figure 2:**
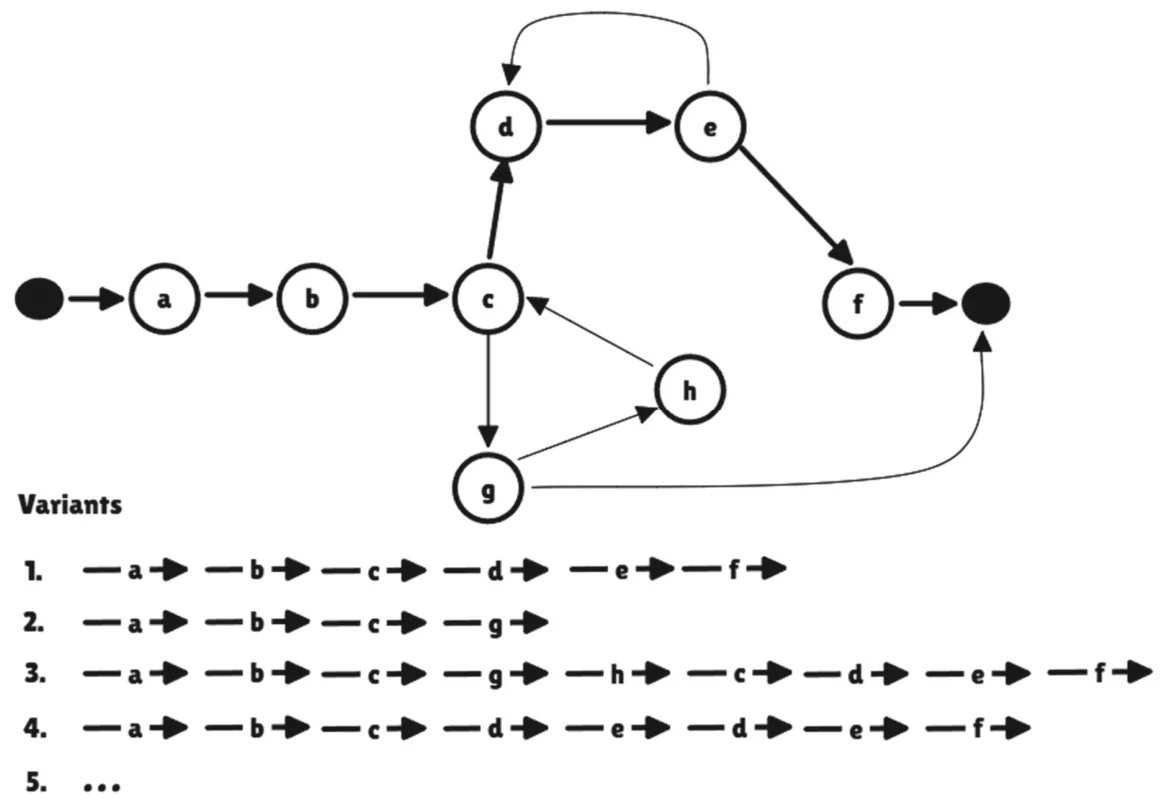
Example of a process map generated from an event log, showing selected alternative process variants.

From the event log, process mining supports three core activities. Discovery infers a process model from the log alone, without assuming any prior model; Figure 2 shows one such discovered model, a process map. Conformance checking compares the log against a reference model and localises deviations. Enhancement extends an existing model using information from the event log, using timing data to identify bottlenecks, resource data to identify handover patterns, or, in the IoT setting, sensor data to correlate physical conditions with process outcomes. In a process map of this kind, nodes represent activities, and arcs represent the transitions observed between them. The thickness of an arc can reflect how frequently that path was traversed, so the dominant route through the process is visible immediately, while node colouring can encode a chosen metric such as frequency or average duration. Because a log holds many variants, the map presents several alternative routes rather than a single sequence. The result is also not a property of the event log alone: different discovery algorithms and different filtering thresholds applied to the same log can yield different models [5].

A discovered process map is therefore only one of many possible views of the event log. The same event log can be mined from several complementary perspectives, each answering a different question about the process. The control-flow perspective reveals what sequence of operations happens. The organisational or resource perspective reveals which machine, sensor, or person handles which steps and how often they hand work off to one another. In an IoT setting, this becomes, in effect, a social network of devices. The time or performance perspective colours the same flow by duration and throughput, thereby exposing bottlenecks. The case perspective follows individual journeys through the system and highlights outliers. Each perspective assumes that the digital event log is in place [5].

### 2.2. Internet of Things—From Sensor Data to Process Events

**Figure 3:**
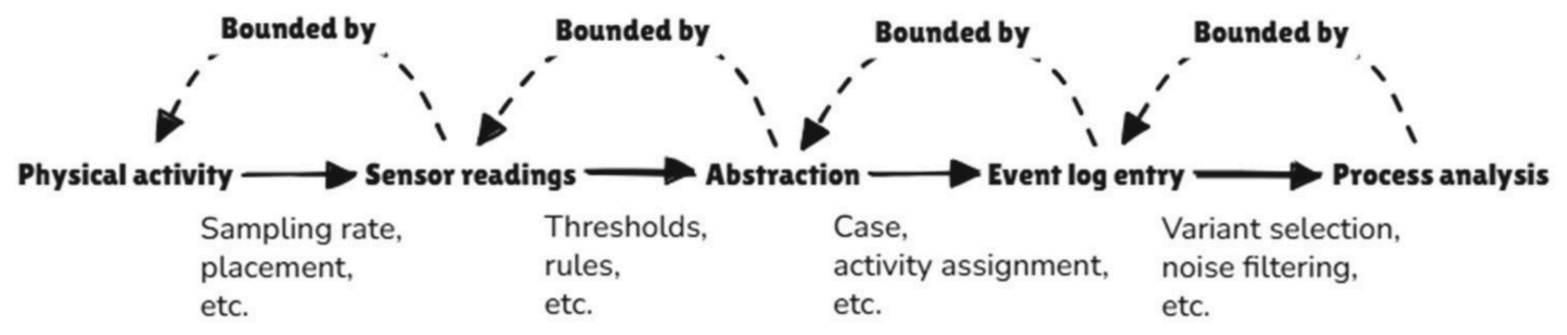
Conceptual representation of the dependency between sensor signals, event abstraction, event logs, and downstream process analysis.

Process mining relies on discrete, activity-level events [5]; sensors emit fine-grained, continuous, low-level signals about motors, switches, temperatures, and vibrations. It is important to note that sensor measurements do not, by themselves, constitute business process event-log entries [2]. Every event-log row that originates from a sensor is the output of some transformation, such as a rule, classifier, threshold, or alignment. The quality of these transformations can therefore have a substantial impact on the quality of downstream process analysis. Figure 3 depicts this dependency as a chain running from the raw signal through the abstraction to the event log and the analysis built on it. The quality of each translation step can therefore affect the quality of the downstream process analysis.

Event abstraction is the process of consolidating multiple fine-grained readings into a single, process-level event. In the absence of this step, the raw data is usually too granular to indicate reliably when a process activity has begun [5,7]. Consequently, the issue has been addressed by several strands of work, which differ in their underlying assumptions. Abstraction, however, is not the first constraint: an earlier choice already limits what abstraction can achieve; both the type of sensor and its placement strongly influence what can be observed and which activities may be distinguishable [11]. Each placement choice can constrain the level of detail and the parts of the process that can be observed before any events have been defined. This creates a circularity with the process-maturity argument developed in Section 4: knowing where to place a sensor presupposes knowing which activities matter, yet that knowledge is often exactly what organisations hope process mining will reveal.

Bridging the gap between low-level IoT sensor signals and high-level process events involves abstraction, contextualisation, and correlation, and researchers have proposed several complementary approaches to make raw sensor data suitable for process mining [13]. Many take the form of multi-stage transformation pipelines that move from preprocessing and activity recognition through to event abstraction [14,15]. Others organise IoT data into event hierarchies, aggregating low-level sensor changes into higher-level process and context events with techniques such as complex event processing or rule-based reasoning [16]. Semantic, ontology-based methods use domain ontologies to map sensor metadata onto process concepts [17]. Interactive, visual analysis helps analysts identify activity signatures when ground truth is missing [18]. Other work integrates AI and large language models, combining deep learning with LLMs for temporal modelling and schema-agnostic parsing [19]. Seiger et al. [13] propose a Complex Event Processing (CEP)-based approach for detecting process activity executions from IoT sensor data at runtime, generating high-level process events for process mining. Their approach addresses limitations of machine learning (ML)-based activity detection, which often relies on specific sensors and trained models, as well as the absence of a central Process-aware Information System (PAIS) in many IoT environments.

Despite these diverse methodological approaches, no single approach appears to have generalised reliably across substantially different domains, as heterogeneity and contextual variability make a universal method difficult to achieve. Different domains, such as smart manufacturing and healthcare, have vastly different sensor modalities, sampling rates, and process semantics; a method that works for discrete movements in a factory may not apply to continuous vital sign monitoring in a hospital. Sensor signals themselves vary significantly, ranging from binary (on/off) to discrete (categorical states) to continuous (time-series), and handling continuous data requires extensive preprocessing and discretisation that may not be necessary for simpler, event-triggered sensors [14]. Beyond these technical challenges, bridging the gap is often nontrivial and error-prone, requiring expert intervention and judgment tailored to the specific analysis goals. Furthermore, abstracting data into high-level event logs can result in the loss of context contained in the original sensor data. Different analysis purposes, such as model discovery and performance monitoring, may also require different levels of granularity. A single fixed abstraction level may therefore be impractical [16].

### 2.3. Research Landscape at the IoT–Process Mining Intersection and Emerging Perspectives

The literature considered in this perspective spans several interconnected research areas, as IoT-enabled process mining brings together concepts from the Internet of Things, process mining, business process management, event data, and process maturity. To capture these different perspectives, relevant literature was identified through keyword-based searches using Google Scholar, IEEE Xplore, and ScienceDirect. The search covered approximately the past ten years, with greater emphasis placed on publications from the most recent three years to capture the current state of research and emerging developments in this rapidly evolving field. The searches combined terms such as “Internet of Things”, “Process Mining”, “Sensor Data”, “Event Data”, “Business Process Management”, and “Process Maturity”, including combinations of these terms where appropriate. Publications were considered relevant when they addressed the use of IoT or sensor data in relation to process mining, business process management, process-event representation, or process and digital maturity. The search focused on English-language publications accessible through the selected academic databases and search engines. Established foundational works were also retained where they provided important conceptual grounding for the perspective.

The identified literature was examined thematically rather than systematically, with attention to the research problems addressed, the role of IoT and sensor data, the capabilities required to use such data for process analysis, and the assumptions underlying these approaches. Recurring concepts, requirements, and capabilities were compared across the identified research streams and consolidated into broader research directions. These directions were then used to position the perspective developed in this paper and to identify candidate assessment areas relevant to IoT-enabled process mining.

The reviewed literature reveals that the IoT–process mining intersection has developed along several complementary research directions. Work on IoT and business process management establishes the broader rationale for connecting physical-world observations with process-level representations [11]. Research on process mining using sensor data focuses more specifically on the technical challenge of transforming continuous and heterogeneous sensor observations into activity-level events [14,15]. Bridging models and semantic approaches address how physical observations can be related to process concepts and event representations [16,17], while interactive and learning-based approaches investigate how activity executions can be detected when explicit event records are unavailable [18,19].

These research perspectives address important but different aspects of the IoT–process mining relationship. The sensor-to-event literature primarily addresses how physical observations can be transformed into process-relevant data, whereas process-mining maturity research [20] addresses how organisations develop the capabilities required to integrate process mining. These perspectives provide important foundations, but neither directly addresses how the maturity of the observed process affects the feasibility and value of connecting physical-world observations to process-level event data. In particular, it remains insufficiently specified under which process conditions adding IoT data to process mining creates sufficient value to justify the additional sensing, abstraction, integration, maintenance, and governance effort.

We use this observation as the starting point for the perspective developed in the remainder of the paper. Rather than treating IoT integration as a single technical problem, we distinguish between two roles that IoT data can play in an event log. IoT can be event-creating, when sensor observations are required to generate events that would otherwise be absent, or event-enriching, when events already exist and sensor data adds contextual information. We then consider how the maturity of the observed process affects the feasibility and potential value of these two pathways.

The resulting perspective connects two aspects that have largely been addressed separately in existing research: the role of IoT data in constructing the process event log and the maturity of the process to which that data is applied. The contribution of this perspective lies not in proposing another sensor-to-event abstraction method or a new maturity model, but in bringing these two aspects into a common analytical view. This allows IoT integration to be considered not only in terms of how sensor observations can be transformed into process information, but also in terms of what role that information plays, what process conditions support its reliable use, and what capabilities may need to be assessed to sustain it. Table 1 summarises the main research directions identified through this synthesis, their primary focus, and how the perspective developed in this paper builds on and extends each direction. This positioning provides the basis for subsequently examining event-creating and event-enriching IoT data, their relationship to process maturity, and the assessment areas that may inform the future development of an IoT-enabled process mining maturity model.

Taken together, the reviewed research provides complementary foundations for understanding the IoT–process mining intersection, from connecting physical-world observations with business processes to transforming sensor data into process events and developing the organisational capabilities required for process mining. The perspective developed in this paper extends these streams by bringing together two aspects that are typically considered separately: the role of IoT data in constructing or enriching the event log and the maturity of the process in which such data is used. This provides a basis for considering not only how IoT data can be integrated into process mining, but also the conditions, capabilities, and requirements that influence the reliability and usefulness of such integration.

## 3. Perspective on the Role of IoT Data in the Event Log

Section 2 examined how sensor signals become events. Before moving forward, however, a clearer distinction is needed regarding the role that IoT data plays once it reaches the event log. IoT data can relate to an event log in two distinct ways: it can be event-creating or event-enriching. When data is event-creating, IoT enables process mining by making events observable where no event records previously existed. When data is event-enriching, IoT acts as an enhancer, providing additional context to events that could already be mined.

**Figure 4:**
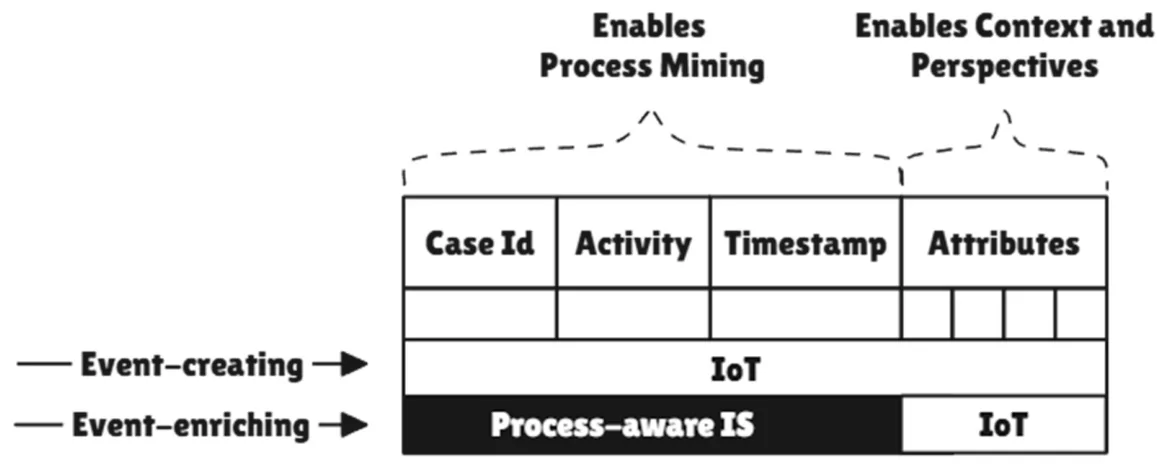
Conceptual representation of event-creating and event-enriching IoT data in relation to the process event log.

Rather than arriving as events, sensor readings are streams of physical measurements that must be interpreted, filtered, and abstracted before they can be used for process-level analysis. This translation is not trivial [11]. A meaningful distinction can be drawn between two pathways. In the first, raw sensor readings are aggregated to create process events that did not previously exist as records. In the second, events already exist in the event log and sensor data is used to enrich them with contextual attributes such as location or machine condition. Figure 4 illustrates these two pathways: in the event-creating pathway, an abstraction step turns the sensor signal into the event log entry itself; in the event-enriching pathway, the log entry originates in a process-aware information system, and the IoT data is attached to it as a contextual attribute. Figure 4 simplifies this in one respect: a process-aware information system typically populates some attribute columns itself, so the IoT contribution is the subset of attributes that such a system cannot observe.

1. In the event-creating pathway, the sensor is the source of the event. Following an abstraction step, the sensor reading becomes the event itself. The data is event-creating: without it, there is no entry in the event log at all. It exists in the log only because some computation decided that a pattern in the raw signal constitutes a process-level occurrence. In this context, IoT therefore provides evidence that may be necessary to make the corresponding event observable in the event log.
2. In the event-enriching pathway, the event already exists from another source, typically a process-aware information system, and sensor data is attached to it as contextual enrichment. The data does not inherently create the event. Removing it leaves the log structurally intact, though it is poorer in context. This enrichment can be implemented at the event level or the case level.

From our perspective, event-creating data poses greater challenges than event-enriching data because the existence of the event log depends on it. The severity of these challenges, however, depends on process maturity. First, the reliability of the approach depends partly on the continued functioning of the sensing infrastructure. It is therefore important to assess sensor redundancy, reading reliability, and the management of physical degradation. Sensors are subject to wear and tear, leading to depreciation, while battery-powered units experience performance decline and must be replaced proactively to prevent failure [7]. When the data is event-creating, such failures can result in missing events in the log. Secondly, the concept of abstraction itself is fragile. How many sensors are required to justify inferring a single activity? The number of sensors required to achieve this varies, and the selection can influence how an event is defined or inferred. Conversely, when an event is enriched with IoT data, these same failures result only in the loss of context, allowing the underlying process to remain observable. This imbalance in risk is, in our view, the main reason for treating the two roles separately.

The classification also points to a future direction. Event-enriching data accumulates alongside events without being responsible for them, so over time it builds into a rich record of contextual data paired with known process occurrences. Once sufficient volumes are available, they can be used for training. The relationship between enriching data and the events it accompanies could be used to develop a machine learning model for predicting or reconstructing events that were never explicitly logged. This could help fill gaps between recorded process steps. The key point is that enriching data through learning can become a source of event creation. Data that began as context can then be used to generate new events and augment the log itself. The two roles should therefore be understood as positions in the data lifecycle rather than fixed categories: the role of a given data stream may shift as the surrounding information systems and models evolve.

### 3.1. Illustrative Example: Event Creation and Enrichment in Smart Manufacturing

The distinction between event creation and event enrichment can be illustrated through a simplified manufacturing process involving the internal delivery of materials to a production workstation. The process consists of four activities: material requested, material picked, material delivered to the workstation, and production started. The example demonstrates that the role of IoT data in process mining depends on the existing level of process instrumentation and standardisation, as sensor data may either create new process events or enrich events that are already available in the event log.

Event-creation scenario: consider a production environment in which the Manufacturing Execution System (MES) records when material is requested and when production starts, but does not explicitly record when the required material is delivered to the workstation. IoT technologies such as RFID tags, indoor positioning systems, or connected devices can continuously or periodically capture the movement and location of materials within the production environment. These observations can be analysed to determine that the material has arrived at the designated workstation. The resulting inference can therefore be transformed into a new process event, for example, Material delivered. In this case, IoT data contributes directly to the construction of the event log by making a previously manual process transition automatic or an unrecorded process transition observable. However, the reliability of such an event depends on the maturity of the underlying process. If material-delivery procedures are not sufficiently standardised, it may be unclear what should constitute Material delivered. For example, the material may be considered delivered when it enters the workstation area, when it is placed at the designated location, or when it is actually made available to the operator. Similarly, if no reliable ground truth exists, it may be difficult to determine whether the inferred event accurately represents the completion of the delivery. Sensor failures, inaccurate location measurements, or incomplete tracking coverage can further result in missing or incorrectly inferred events. Consequently, when IoT data is used to create events, weaknesses in process standardisation, event definitions, measurement reliability, or validation mechanisms can directly affect the completeness and correctness of the resulting event log.

Event-enrichment scenario: now consider the same production environment in which the MES already records the event Material delivered. IoT technologies can provide additional contextual information associated with this event, such as the material’s location, delivery time or transport duration. Rather than creating a new event, the sensor data enriches an existing event with additional attributes describing the circumstances of the material delivery. The resulting event may therefore contain not only the activity and timestamp, but also contextual information that can subsequently be used to analyse material-flow efficiency, waiting times, production delays, or differences between production cases. Because the core process event is already recorded by the MES, a failure of the IoT tracking system does not necessarily eliminate the event from the event log. Instead, the failure primarily results in the loss or degradation of contextual information. The process model can therefore remain structurally observable even when some IoT measurements are unavailable. This makes event enrichment less dependent on IoT data for basic event-log completeness, although the value of the resulting analysis still depends on the quality, temporal alignment, and consistency of the additional sensor data.

These two scenarios illustrate an important distinction for IoT-enabled process mining. When IoT data creates events, sensor observations become part of the mechanism through which process execution is reconstructed. Consequently, process standardisation, event definitions, ground truth, and sensing reliability become important considerations for obtaining a trustworthy event log. When IoT data enriches existing events, the underlying process execution is already represented in the event log, while IoT data primarily increases its contextual richness.

### 3.2. Operational Criteria for Distinguishing Event Creation and Event Enrichment

To make the distinction more explicit and practically applicable, Table 2 summarises a set of operational criteria for differentiating event-creating and event-enriching IoT data. The criteria consider the origin of the event, the dependency of the event log on IoT data, the required level of abstraction, the consequences of data or sensor failure and the validation requirements. These criteria are intended as practical indicators rather than strict classification rules, as the role of IoT data may depend on the existing process instrumentation and may change as the surrounding information systems evolve.

These criteria provide a practical basis for classifying the role of IoT data in an event log. In particular, the key distinction is whether IoT data is necessary to establish the existence of the event itself or instead provides additional information about an event that is already observable.

## 4. Perspective on the Impact of Process Maturity for IoT-Enabled Process Mining

The availability of an event log indicates that at least some aspects of a process have been instrumented and recorded, but it does not by itself imply that the underlying process is stable, well-defined, or consistently executed. This distinction is particularly relevant when introducing IoT data into process mining. IoT sensing can increase the observability, granularity, and contextual richness of process executions, but additional observations do not necessarily translate into more useful process knowledge. Their usefulness depends, among other factors, on whether process behaviour can be interpreted consistently and whether the resulting data are sufficiently reliable for the intended analysis [23,24,25].

Process maturity is therefore considered as one contextual factor that may influence the usefulness of IoT-enabled process mining. Here, maturity refers to the extent to which a process is defined, managed, measured, controlled, and consistently applied. It should not, however, be interpreted as a direct predictor of analytical capability or business value. Rather, it provides a way of characterising the extent to which the process and its supporting practices have become institutionalised and measurable.

Maturity has been conceptualised through staged models across several domains. An influential early example is the Capability Maturity Model (CMM), developed at the Software Engineering Institute, which conceptualises process capability as a progression from ad hoc and reactive practices towards quantitatively managed and continuously improving practices [26]. Building on this broader tradition, maturity models have also been developed specifically for business process management. The Business Process Maturity Model (BPMM), formalised by the Object Management Group (OMG), conceptualises process maturity as an evolutionary progression through five levels, reflecting increasing degrees of process standardisation, management, measurement, control, and continuous improvement [27]. However, neither CMM nor BPMM specifies the characteristics of sensor-based process observation, event generation, or the use of IoT data for process mining. They are therefore used here as conceptual foundations for understanding process maturity, rather than as validated maturity models for IoT-enabled process mining.

A similar staged progression can be observed in commonly distinguished forms of analytics, from descriptive and diagnostic towards predictive and prescriptive applications [28]. The two hierarchies should not be interpreted as directly corresponding to one another. Process maturity describes the development of process-related capabilities, whereas the analytics hierarchy describes increasing analytical sophistication. Both can be understood as cumulative progressions in which capabilities established at earlier stages may provide a foundation for later ones.

The difficulty of transforming physical observations into reliable process events suggests that the value of IoT-enabled process mining may be contingent on process maturity. Where process concepts, activities, and relevant states are poorly defined, additional sensing may increase data availability without proportionally increasing process knowledge [29]. A process may also be organisationally mature while lacking the sensing infrastructure required to observe relevant physical activities [22,30]. Conversely, IoT devices may provide extensive observations without the process being sufficiently defined or the data sufficiently contextualised for meaningful analysis. Existing process mining maturity models already recognise the importance of the underlying data foundation. For example, Brock et al. [20] identify Data Foundation as one dimension of process mining maturity, including aspects such as process-oriented information systems, data accessibility, and data scope.

The IoT setting introduces additional characteristics that may need to be considered within such a data foundation. Sensor-based observation creates a dependency between the physical process and the resulting event data: whether an event can be observed, when it can be observed, and how accurately it can be represented are influenced by the sensing infrastructure as well as on the process itself [19]. Consequently, event observability, sensor accuracy, data completeness, temporal resolution, contextual richness, and process variation can be considered potential indicators of the maturity of an IoT-enabled process-data foundation. These are not proposed as a complete maturity model, but as observable characteristics that may be relevant when assessing whether an IoT-enabled process can provide reliable process-mining data.

The relevance of these characteristics also depends on the role of IoT data within the event log, as discussed in Section 3. When IoT data is event-creating, sensor observations are used to establish process events themselves, making the observability and reliable interpretation of relevant physical states particularly important. When IoT data is event-enriching, the underlying events may already be available and sensor observations instead provide additional context, making factors such as temporal alignment, contextual relevance, and data quality particularly important. Thus, IoT-specific data requirements depend not only on process maturity but also on how sensor data are used within the event log.

Several process characteristics may be relevant when considering the role of process maturity in IoT-enabled process mining. Table 3 summarises illustrative characteristics that may be considered in this context. Their relationship with process maturity should not be assumed to follow a predetermined direction, as their relevance and development may depend on the process context and assessment objective.

Greater maturity may itself require additional documentation, monitoring, infrastructure, and organisational effort, while stronger governance may increase operational costs while improving traceability or compliance. The value of IoT-enabled process mining, however, cannot be reduced to operational performance alone. In some processes, relevant outcomes may include throughput, waiting time, resource consumption, or cost, while in regulated or safety-critical settings the primary benefits may instead concern traceability, compliance, auditability, or reconstruction of past events. IoT instrumentation may therefore be justified even when no direct performance improvement is expected. At the same time, sensing introduces its own ongoing requirements, including deployment, calibration, maintenance, data-quality management, and model validation. Processes may also change over time due to regulatory, organisational, technological, or operational changes [21,31].

The above considerations suggest that process maturity should be viewed as one contextual dimension of IoT-enabled process mining rather than as a direct predictor of business value. Existing maturity models provide useful foundations for describing general process capability and process-mining readiness [20,26], but they do not necessarily capture the characteristics introduced when process events are generated or enriched through physical sensing. An IoT-specific maturity perspective could therefore extend these existing approaches by explicitly considering the capabilities required to observe, transform, validate, integrate, and maintain sensor-derived process information. Such a perspective could provide a structured means of assessing which aspects of a process are observable and which can be measured reliably. It could also help determine which forms of IoT-enabled process analysis can reasonably be supported, while recognising that the appropriate level of sensing and analytical sophistication depends on the specific process and its objectives.

## 5. Considerations for the Development of an IoT-Enabled Process Mining Maturity Model

A maturity model can provide a structured approach for assessing and developing the capabilities required to achieve a particular organisational or technological objective. It can identify relevant assessment areas, define the capabilities and practices associated with these areas, and provide measurable indicators for evaluating the current state and identifying capability gaps. In this way, maturity assessment can support not only the classification of a current state but also the identification of areas for improvement [20,22,26]. In the context of IoT-enabled process mining, this perspective can help structure the capabilities required to obtain reliable process knowledge from IoT data. Based on the considerations developed in the previous sections, the following assessment areas are proposed as a conceptual basis for the future development and empirical validation of an IoT-enabled process mining maturity model. These candidate assessment areas are summarised in Table 4, together with illustrative aspects that could be considered within each area.

A first consideration concerns the reliability of the IoT-to-event-log transformation. As outlined in Section 2.2, the quality of an IoT-enabled event log depends not only on the original sensor measurements but also on the transformations through which physical observations are converted into process events or event attributes. Potential assessment aspects therefore include sensor reliability, data completeness, temporal accuracy, event observability, abstraction validity, semantic consistency, and traceability of event-generation decisions.

A second consideration concerns the role of IoT in process observability. Based on the findings reviewed in Section 3, IoT data may be distinguished as either event-creating or event-enriching. In the former case, sensor observations contribute directly to establishing process events, whereas in the latter they provide additional context to events that are already available. These roles imply different requirements for process observation and event generation. A future maturity assessment should therefore consider not only whether IoT data are available, but also what role they play in making process execution observable and how comprehensively relevant process behaviour is captured.

A third consideration concerns the readiness of the underlying process and data environment. As argued in Section 4, process maturity describes the extent to which a process is defined, measured, controlled, and consistently applied, while existing process mining maturity models also emphasise the importance of a data foundation [20]. For IoT-enabled process mining, this may involve assessing aspects such as the stability and variation in the process, the coverage of existing event logs, the availability of relevant data sources, and the extent to which existing process and information systems can support the intended IoT-enabled analysis.

A fourth consideration concerns sensing and infrastructure readiness. IoT-enabled process mining may rely on infrastructure that already exists for operational monitoring or control, or it may require dedicated instrumentation designed specifically to improve process observability. Existing infrastructure may provide an opportunity for reuse, but its measurements should first be shown to be meaningfully related to process activities. Purpose-built instrumentation, in contrast, may provide greater control over what is observed, while introducing additional requirements for deployment, integration, calibration, maintenance, and governance. A future maturity assessment could therefore consider the ability to identify, integrate, maintain, and appropriately configure sensing infrastructure for process-mining purposes.

Finally, the value and feasibility considerations are relevant when assessing the suitability of an IoT-enabled process mining approach. IoT deployments can require substantial technical and organisational effort, including sensor acquisition and installation, connectivity, data processing, integration, maintenance, and calibration. The justification for this effort depends on factors such as the importance of currently unobservable process behaviour and the expected analytical, operational, compliance, or traceability value of improved observability. These factors should, however, be distinguished from maturity itself. Expected value and instrumentation cost are better treated as contextual decision criteria than as direct indicators of organisational maturity.

These areas should not yet be interpreted as a definitive set of maturity dimensions, but as a starting point for future assessment and empirical validation. Future research should examine their distinctiveness, operationalisation, and suitability for defining maturity levels, while distinguishing maturity indicators from contextual factors such as expected value and deployment effort. The resulting maturity perspective should therefore focus not simply on technological sophistication, but on whether the capabilities and conditions required to obtain trustworthy process knowledge from IoT data are in place, including process and data readiness, sensing capabilities, and the reliability of the physical-to-process abstraction.

## 6. Discussion

Turning a signal into an event is a well-recognized research and technical problem, and a range of approaches to addressing it exists (e.g., developing more sophisticated pipelines, better machine learning models, and richer semantic enrichment), as reviewed in Section 2.2. That effort, however, entails technical and organisational commitments: sensor deployment, continuous data preprocessing, and ongoing abstraction maintenance are recurring commitments. We therefore argue that whether and when IoT integration is justified should be considered before how such integration should be implemented. This motivates the need for structured guidance to assess which processes and process contexts may justify IoT integration. The following sections outline what such guidance would need to address. More specifically, such an assessment needs to consider both the role that IoT data plays in the event log and the conditions under which the process can effectively support its use. As developed in Section 3, IoT data may be event-creating, where sensor observations are transformed into events that would otherwise be unavailable, or event-enriching, where sensor data provides additional context to events already captured by other systems. In parallel, as discussed in Section 4, process maturity provides a complementary perspective for considering whether the process is sufficiently defined, managed, measured, and controlled to support the reliable and sustained use of IoT-enabled process mining.

### 6.1. The Relationship Between IoT Data Roles and Process Maturity

Event-creating and event-enriching data represent two different pathways to the event log, but the feasibility and value of each pathway depend partly on how clearly the underlying process is defined and how reliably its execution can be observed. When IoT data is used to create events, physical observations must be mapped onto process-level concepts. This requires more than accurate sensing: the process benefits from sufficiently stable definitions of activities, boundaries, and expected states for observations to be interpreted consistently. Process maturity can therefore influence the reliability with which sensor observations can be abstracted into process-level events. Where process execution is highly variable or poorly defined, the same sensor observation may support different interpretations, making it more difficult to determine what should constitute an event and when that event has occurred. As a result, lower process maturity may increase the uncertainty associated with event-creating IoT data.

Event-enrichment has a different dependency. Because the underlying event is already available from a process-aware information system, IoT data does not need to establish the basic observability of process execution. This makes enrichment less dependent on IoT data for event-log completeness. Nevertheless, process maturity may influence the extent to which enrichment can be interpreted and used effectively. If the process is insufficiently defined or unstable, additional contextual attributes may be difficult to relate to meaningful process behaviour. Higher maturity can therefore increase the analytical value of enrichment by providing a stable process context against which sensor-derived characteristics can be compared.

The two roles should therefore not be understood as simply corresponding to low and high process maturity. A mature process may still contain activities that are invisible to conventional information systems and consequently require IoT-based event creation. Conversely, IoT enrichment may provide useful contextual information even when a process is only partially defined. Process maturity is better understood as a set of conditions that influence the reliability and usefulness of each IoT role rather than as a binary prerequisite for IoT integration.

The relationship between IoT data roles and process maturity can therefore be conceptualised as a bidirectional feedback relationship with potentially reinforcing effects. Process maturity influences the reliability with which IoT observations can be transformed into events or meaningfully associated with existing events, while IoT-enabled observability can generate process knowledge that supports the clarification, standardisation, and refinement of process execution. This creates a potential feedback loop in which greater process maturity can improve the effectiveness of IoT integration, while improved IoT-enabled observability can contribute to further process development. The strength and direction of these effects may depend on the specific process context and on how IoT observations are incorporated into process management practices. This leads to a broader view in which process maturity and the role of IoT data form two interacting perspectives.

### 6.2. A Structured Space of Open Topics and Questions

Taken together, the two perspectives define a space of open topics whose axes are the role that the data plays and the maturity of the observed process. The topics that follow are neither exhaustive nor uniformly applicable. Which of them matter, and what counts as an answer, depends on the process and its context.

#### 6.2.1. The Challenge of Undefined Processes

In an undefined process, a central challenge arises regardless of whether sensor data creates or enriches events: without a stable process context, there is little basis for specifying what counts as an event, how cases should be identified, or what enrichment would even be meaningful. When a reference model is unavailable, validation against a predefined process model may not be possible. However, independent ground truth can still be established through direct observation, expert annotation, or controlled experimentation. The absence of a reference model may therefore increase the difficulty of validating whether inferred events accurately represent process execution, particularly when independent validation data are also unavailable. The reliability of the underlying process is also a concern, as neither creation nor enrichment can compensate for an inaccurate source of records. However, the consequences tend to be more severe in the event-creating pathway, since the log depends on sensor data abstraction, so flaws in the abstraction can propagate directly into the event log. In the event-enriching pathway, the log already exists from a process-aware information system, so enriching existing events with IoT attributes requires less additional work and enrichment can be added incrementally where suitable data is available.

#### 6.2.2. The Challenge of Sustaining Value

Once a process is defined and measured, the challenges shift from basic feasibility to sustaining and justifying IoT integration. Both pathways address sensor reliability, maintenance overhead, and the need to distinguish meaningful signals from noise or artefacts. In the event-creating pathway, these concerns are more consequential: sensor failures, drift in abstraction rules, or misclassification can result in missing or incorrectly generated events in the log. Sensor type and placement can constrain the observable process boundaries and granularity available for subsequent analysis. Distinguishing a genuine conformance deviation from a sensing artefact requires careful diagnostics, as sensing artefacts can appear as false positives. In the event-enriching pathway, the risks tend to be lower because removing enriched data leaves the log structurally intact. In our view, the central question shifts to optimisation: it remains open which contextual attributes measurably improve enhancement outcomes, and how organisations should determine what adds value. Accumulated enriched data can also serve as a training corpus for learned event abstraction, potentially transforming enrichment into a source of event creation over time.

#### 6.2.3. Cross-Cutting Considerations

Regardless of maturity or data role, several issues persist. Benefit thresholds would need to be established to determine what measurable criteria justify sustained instrumentation, given the costs of deployment and maintenance. Domain dependence complicates matters, as, to our knowledge, no abstraction method has yet generalised across contexts. Governance and privacy arise wherever sensor-derived logs concern people [32]. The event-creating pathway requires an abstraction layer to generate the event log from observations. This generally requires some prior specification of the relevant process-level events, which may be more difficult when the process is poorly defined. This is the circularity identified in Section 2.2. The event-enriching pathway is more flexible as events originate from process-aware information systems and sensor context, which can be added incrementally at either the event or case level.

### 6.3. Future Outlook: Autonomous Systems and Accountability

As IoT-enabled systems become more autonomous, the role of sensor data in process observation is likely to become increasingly important. Autonomous robots and other cyber-physical systems can generate large volumes of process-relevant observations, potentially extending process mining beyond information already captured by conventional information systems [3,4]. In this context, the distinction between event-creating and event-enriching IoT data introduced in Section 3 becomes particularly relevant. Sensor data may not only provide additional context for known process events, but may also make previously unobservable activities, states, or transitions visible.

This development also raises questions about the role and quality of the resulting event logs. As discussed in Section 2 and Section 3, transforming sensor observations into process-level events requires decisions about observability, temporal accuracy, abstraction, and semantic interpretation. As autonomous systems increasingly act on sensor-derived information, the reliability and traceability of these transformations may become important not only for process analysis, but also for reconstructing how a process evolved. They may also help explain how particular decisions were reached. Process mining can provide a structured representation of these observations, but the usefulness of such a representation depends on the quality and maturity of the underlying IoT-to-event-log pipeline.

This creates a further research direction for the maturity perspective introduced in Section 4 and Section 5. Future IoT-enabled process mining maturity models could consider the extent to which sensor observations are systematically captured, transformed, validated, and maintained as process-relevant event data. Such capabilities may become increasingly important in autonomous settings, where human actors may no longer directly observe or document individual process decisions. The EU Artificial Intelligence Act already requires certain high-risk AI systems to support automatic event logging and imposes requirements concerning the retention of such logs [33]. However, these requirements primarily concern the logging of the AI system itself. It remains an open question how such machine-level records should relate to the process-level event logs considered in this article, particularly when IoT observations contribute to creating or enriching those events.

Accordingly, future research could investigate how IoT observability, event-log quality, process maturity, and accountability can be considered together. An auditable event log would not, by itself, establish responsibility for an autonomous decision, but it could provide an important evidentiary basis for reconstructing the process context in which that decision occurred.

## 7. Limitations

The findings and proposed perspective should be interpreted in light of the following limitations. First, the proposed perspective is conceptual and has not been empirically validated. The relationship between the role of IoT data in the event log and process maturity is therefore based on the synthesis and interpretation of existing research. The assessment areas presented in Section 5 represent candidate areas for a future maturity model, while their measurability, distinctiveness, and relationships remain to be examined empirically.

Second, the literature was identified through keyword-based searches and examined thematically. The review was intended to establish the conceptual foundations of the perspective and was not designed as a systematic or exhaustive literature review.

Third, the perspective is intentionally domain-independent. The characteristics of IoT-enabled process mining can vary substantially across application domains because sensor technologies, process structures, event definitions, and analytical objectives differ. The proposed relationships and assessment areas may therefore require adaptation to specific contexts.

Finally, the paper does not establish quantitative criteria for determining when IoT integration is justified. The potential value of additional observability depends on the process, the analytical objective, the available infrastructure, and the associated deployment and maintenance effort. Establishing measurable decision criteria and examining their relationship with process maturity remains an open area for future research.

## 8. Conclusions

This paper presents a perspective on IoT-enabled process mining centred on two connected dimensions: the role of IoT data in the event log and the maturity of the process being observed. The distinction between event-creating and event-enriching IoT data shows that sensor data can contribute to process mining in fundamentally different ways. Event-creating data provides evidence for process events that would otherwise be unavailable, making sensing, abstraction, and validation directly relevant to event-log reliability. Event-enriching data adds contextual information to events already captured by other systems, with the main requirements concerning data quality, temporal alignment, and contextual relevance.

Process maturity provides a complementary perspective for assessing these roles. The extent to which a process is defined, stable, measurable, and supported by appropriate data and infrastructure influences the reliability and usefulness of IoT-enabled process analysis. At the same time, increased physical observability can contribute to process understanding by exposing previously unrecorded activities, variation, and contextual factors. The perspective developed in this paper identifies a set of candidate assessment areas for an IoT-enabled process mining maturity model. These areas address process observability, IoT-to-event-log reliability, process and data readiness, sensing and infrastructure readiness, and the value and feasibility of additional instrumentation. They provide a conceptual basis for future empirical work on operationalising and validating such a model.

Future research should examine these relationships across different domains and process conditions, establish measurable maturity criteria, and investigate how IoT-enabled event logs can support increasingly autonomous process execution. In particular, reliable and traceable transformation from physical observations into process-level information may become increasingly important for process analysis, auditability, and accountability.

## Figures and Tables

**Table 1:** Positioning of the perspective in relation to existing research at the IoT–process mining intersection.

Research Direction	Main Focus	Perspective Contribution
IoT and business process management [11].	Connecting physical-world data with business processes.	Adds the role of IoT data and process maturity
Sensor-based process mining [14,15].	Transforming sensor observations into process-level events.	Distinguishes event creation from event enrichment and considers their different reliability and validation requirements
Bridging Model and semantic approaches [16,17].	Relating physical observations to process concepts and event representations.	Considers abstraction as part of a broader IoT-to-event-log capability rather than as an isolated technical problem.
Interactive and learning-based event detection [18,19].	Detecting activities when explicit event records are unavailable.	Considers the implications of event creation for event-log completeness, reliability, and validation
Process-mining maturity [20].	Organisational and analytical capabilities for exploiting process mining.	Connects process maturity with IoT-based observability
IoT and digital readiness/maturity [21,22]	Assessing readiness for digitalisation and IoT adoption.	Extends readiness toward IoT-enabled process mining

**Table 2:** Operational criteria for distinguishing event-creating and event-enriching IoT data.

Criterion	Event-Creating	Event-Enriching
Source of event	IoT data provides the primary evidence from which the event is inferred.	The event originates from an existing process-aware information system or other event source.
Event-log dependency	Without the IoT-derived observation, the corresponding event is absent from the log.	The event remains in the log without the IoT data, although contextual information is lost.
Abstraction requirement	Raw sensor observations must be abstracted into a process-level event.	Sensor observations are associated with an already defined event or case as additional attributes.
Failure consequence	Sensor or inference failures can cause missing or incorrectly generated events.	Sensor failures primarily cause missing or degraded contextual information.
Validation requirement	Requires validation that the inferred sensor pattern corresponds to the intended process occurrence.	Primarily requires validation of data quality, temporal alignment, and the relationship between sensor data and existing events.

**Table 3:** Illustrative process characteristics that may be considered when assessing process maturity in the context of IoT-enabled process mining.

Process Characteristic	Assessment Considerations
Process performance	Consistent definitions and measurement of process performance may facilitate monitoring and analysis. The relevant measures depend on the characteristics and objectives of the process.
Governance and control	Defined responsibilities, controls, and monitoring practices may support systematic process observation, management, and traceability.
Operational risk	Systematic identification and management of deviations may support more reliable process monitoring, while increasing process complexity may introduce additional dependencies and risks.
Cost per cycle	Consistent attribution of resources and costs may support the analysis of economic outcomes. At the same time, sensing, monitoring, infrastructure, and governance may introduce additional costs.
Traceability and compliance	In regulated or safety-critical contexts, structured process management may support systematic reconstruction, monitoring, and auditing of process execution.

**Table 4:** Candidate assessment areas for the development of an IoT-enabled process mining maturity model.

Assessment Area	Illustrative Assessment Aspects
IoT-to-event-log reliability	Sensor reliability, data completeness, temporal accuracy, event observability, abstraction validity, semantic consistency, traceability.
IoT role and process observability	Event-creating vs. event-enriching data, event coverage, observability of relevant process states.
Process and data readiness	Process definition, stability and variation, existing event-log coverage, data availability.
Sensing and infrastructure readiness	Existing vs. purpose-built infrastructure, sensor configuration, integration, connectivity, maintenance.
Value and feasibility	Expected analytical, operational, compliance, or traceability value; instrumentation effort, cost, and governance requirements.

## Data Availability

No new data were created or analyzed in this study. Data sharing is not applicable to this article.

## Abbreviations

The following abbreviations are used in this manuscript:

IoT	Internet of Things
CMM	Capability maturity model
BPMN	Business Process Model and Notation
PAIS	Process Aware Information System
CEP	Complex Event Processing
BPM	Business Process Management
ML	Machine Learning
LLM	Large Language Model
MES	Manufacturing Execution System
AI	Artificial Intelligence
BPMM	Business Process Maturity Model
